# Hypoxic Preconditioning Inhibits Hypoxia-induced Apoptosis of Cardiac Progenitor Cells via the PI3K/Akt-DNMT1-p53 Pathway

**DOI:** 10.1038/srep30922

**Published:** 2016-08-04

**Authors:** Rongfeng Xu, Yuning Sun, Zhongpu Chen, Yuyu Yao, Genshan Ma

**Affiliations:** 1 Department of Cardiology, Zhongda Hospital, Medical School of Southeast University, Nanjing 210009, Jiangsu, China

## Abstract

Research has demonstrated that hypoxic preconditioning (HP) can enhance the survival and proliferation of cardiac progenitor cells (CPCs); however, the underlying mechanisms are not fully understood. Here, we report that HP of c-kit (+) CPCs inhibits p53 via the PI3K/Akt-DNMT1 pathway. First, CPCs were isolated from the hearts of C57BL/6 mice and further purified by magnetic-activated cell sorting. Next, these cells were cultured under either normoxia (H0) or HP for 6 hours (H6) followed by oxygen–serum deprivation for 24 hours (24h). Flow cytometric analysis and MTT assays revealed that hypoxia-preconditioned CPCs exhibited an increased survival rate. Western blot and quantitative real-time PCR assays showed that p53 was obviously inhibited, while DNMT1 and DNMT3β were both significantly up-regulated by HP. Bisulphite sequencing analysis indicated that DNMT1 and DNMT3β did not cause p53 promoter hypermethylation. A reporter gene assay and chromatin immunoprecipitation analysis further demonstrated that DNMT1 bound to the promoter locus of p53 in hypoxia-preconditioned CPCs. Together, these observations suggest that HP of CPCs could lead to p53 inhibition by up-regulating DNMT1 and DNMT3β, which does not result in p53 promoter hypermethylation, and that DNMT1 might directly repress p53, at least in part, by binding to the p53 promoter locus.

Despite progress in coronary heart disease therapy, including drug treatments, percutaneous coronary intervention, coronary artery bypass grafting and heart transplantation, congestive heart failure (CHF) after acute myocardial infarction (AMI) remains a leading cause of morbidity and mortality worldwide^1^,^2^. Stem cell therapy, particularly cardiac progenitor cell (CPC) transplantation, maybe a promising novel approach for treating patients with advanced heart failure caused by AMI. Among these CPCs, c-kit-positive CPCs exhibit enhanced proliferation and differentiation abilities to repair injured myocardium and are the most promising candidates for cell therapy for CHF^3^,^4^.

Regardless of the significant advances in cell therapy, the poor survival of transplanted CPCs limits the effectiveness of stem/progenitor cell therapy^5^,^6^. Therefore, effective methods must be identified to promote progenitor cell survival and long-term engraftment after transplantation. CPCs are preconditioned with exogenous stimuli to adapt to the harsh, low oxygen tension environment in ischaemic heart tissue. Previous reports from our group and others have demonstrated that hypoxic preconditioning (HP) with sublethal hypoxic insult can enhance the ability of stem cells to survive and proliferate *in vitro* and *in vivo* after transplantation^7^,^8^,^9^. However, the mechanisms underlying these protective effects are not fully understood.

The phosphoinositide 3-kinase (PI3K)/Akt pathway is activated in response to numerous endogenous and exogenous stimuli. As a critical regulator of PI3K-mediated cell survival, constitutive activation of Akt signalling is sufficient to block cell death induced by a variety of apoptotic stimuli. Many reports have demonstrated that the pro-survival function of Akt is activated as a mediator of the preconditioning signal by hypoxia in various cell types^10^,^11^. Furthermore, previous studies have suggested that HP inhibits apoptosis in rat myocytes through Akt activation^12^.

p53 is a well-known pro-apoptotic tumour suppressor gene; its role has been well documented in cancer research^13^. Many studies in recent years have indicated that p53 activation plays a critical role in damaged myocardial tissue caused by hypoxia and ageing^14^. Furthermore, p53 expression in the heart is up-regulated by the stresses that cause CHF, particularly ischaemia^15^. However, recent studies have demonstrated that HP induces p53 suppression through hypoxia-inducible factor-1α^16^. Moreover, p53 suppression and mitochondrial inhibition may be involved in the cytoprotective effects of HP^17^.

DNA methylation is an important epigenetic modification for gene silencing, with S-adenosyl methionine (SAM) serving as a methyl donor. DNA methylation is catalysed by a family of DNA methyltransferase (DNMT) enzymes, namely, DNMT1, DNMT3α, and DNMT3β. DNMT1 is a maintenance-type methyltransferase that is responsible for maintaining the methylation pattern of the genome in daughter cells during cell division, whereas DNMT3α and DNMT3β are essential for *de novo* methylation^18^. Previous reports have demonstrated that hypoxia could increase DNMT expression and induce global DNA hypermethylation, which play important roles in modulating hypoxia-induced fibrosis within the heart^19^. Furthermore, several groups have recently reported that DNMT1 induces gene repression without the need for its catalytic activity^20^,^21^, although the importance of its methyltransferase function was undeniable.

However, the roles and catalytic activity of DNMTs in p53 modulation of hypoxia-preconditioned CPCs remain unclear. The present study reports that HP of CPCs represses p53 by activating the PI3k/Akt pathway and up-regulating DNMT1 and DNMT3β. This action does not result in p53 promoter hypermethylation. In addition, DNMT1 might directly repress p53, at least in part, by binding to the p53 promoter locus.

## Results

### CPC generation and phenotypic characterization

**Figure 1:**
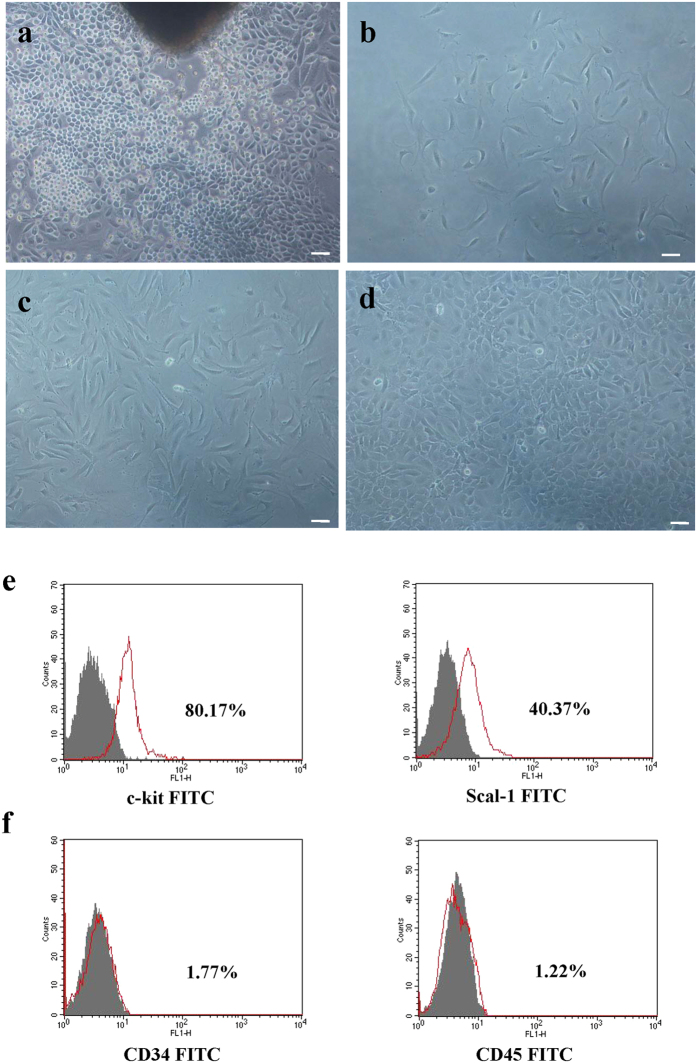
Characterization of cultured CPCs. (**a**) Cells (small, round, phase-bright) migrated from the cardiac explants, aggregated and proliferated over the fibroblast layer after 10 days of culture. Scale bar: 50 μm. (**b**) Phase-contrast image of CPCs after magnetic-activated cell sorting for 1 day. (**c**) Phase-contrast image of CPCs after magnetic-activated cell sorting for 3 days. (**d**) Representative clone generated by CPCs after magnetic-activated cell sorting for 7 days. (**e**) Representative flow cytometric analyses of CPCs for positive expression of the cell surface markers c-kit and Sca-1. (**f**) Representative flow cytometric analyses of CPCs for negative expression of the cell surface markers CD34 and CD45.

CPCs were obtained by mild enzymatic digestion of adult C57BL/6 mouse hearts. A layer of fibroblast-like cells emerged from adherent myocardial tissues followed by small, round, phase-bright cells after approximately 10 days of culture (Fig. 1a). CPCs were then separated by magnetic-activated cell sorting using c-kit magnetic beads and allowed to grow in cardiosphere growing medium. Images of c-kit (+) CPCs were obtained using an inverted phase-contrast microscope after magnetic-activated cell sorting for 1 and 3 days (Fig. 1b,c). These cells presented clone-like proliferation after magnetic-activated cell sorting for 7 days (Fig. 1d). These cells were identified by flow cytometric analysis of the following cell surface markers: c-kit (80.17%), Sca-1 (40.37%), CD34 (1.77%) and CD45 (1.22%) (Fig. 1e,f).

### Cytoprotective effects of hypoxic preconditioning via the PI3K/Akt pathway

**Figure 2:**
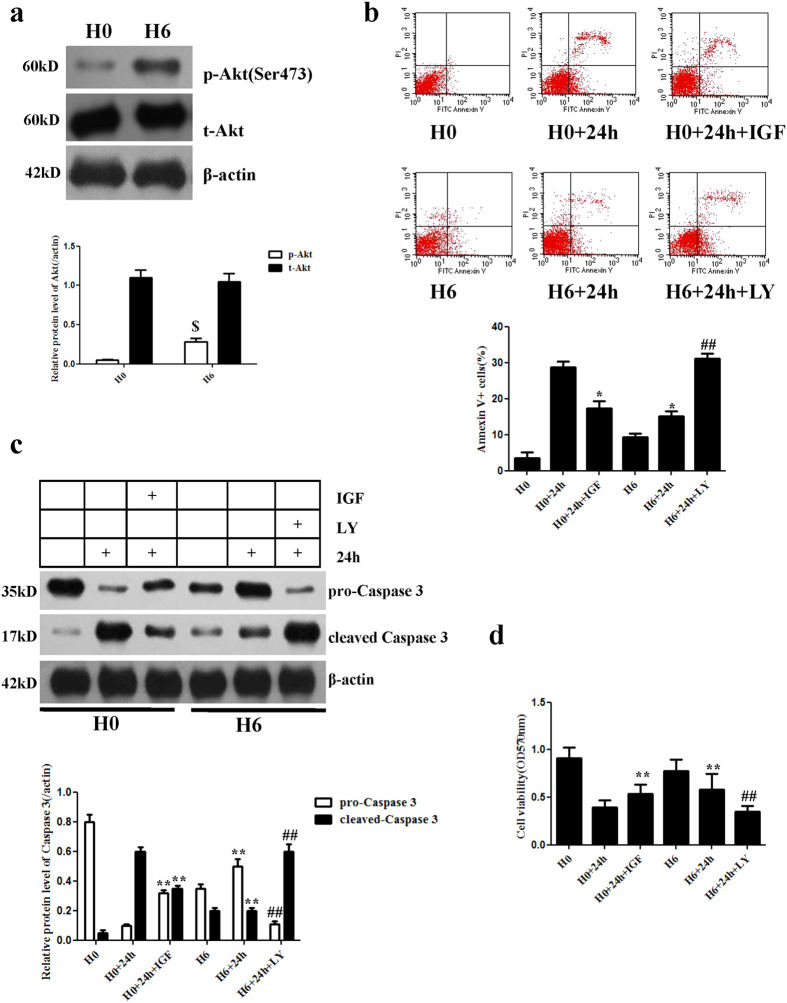
Effects of hypoxia preconditioning on CPCs. (**a**) Representative Western blots of Akt activation by HP. β-Actin was used as a loading control. p-Akt expression was significantly up-regulated in hypoxia-preconditioned CPCs. (**b**) Representative flow cytometric analysis and quantitative analysis of apoptotic cells after the cells were labelled with annexin V and propidium iodide. The apoptosis rate in hypoxia-preconditioned CPCs was markedly lower than in the untreated group. (**c**) Representative Western blots of caspase 3 protein expression in CPCs. The expression level of cleaved caspase 3 was decreased by HP. (**d**) Quantitative analysis of cell viability by MTT assay. Data were obtained from three independent experiments under the same experimental conditions and are expressed as the mean ± SD. ^$^P < 0.01 vs. H0. *P < 0.05 vs. H0+24 h. **P < 0.01 vs. H0+24 h. ^##^P < 0.01 vs. H6+24 h. H0, normoxia. H0+24 h, normoxia + oxygen–serum deprivation for 24 hours. H0+IGF+24 h, normoxia with IGF-1 + oxygen–serum deprivation for 24 hours. H6, HP for 6 hours. H6+24 h, HP for 6 hours + oxygen–serum deprivation for 24 hours. H6+LY+24 h, HP for 6 hours with LY294002 + oxygen–serum deprivation for 24 hours.

Our group previously reported that HP of c-kit (+) CPCs for 6 hours was the optimal time course for protective effects^7^,^8^; therefore, we choose 6 hours of HP as the treatment condition. These cells were cultured under normoxic or hypoxic (0.1%O_2_) conditions prior to oxygen–serum deprivation for 24 hours (24 h). We assessed hypoxia-associated protein expression by Western blot and found that phosphorylated Akt (p-Akt) expression was significantly up-regulated in CPCs that underwent HP for 6 hours (H6) compared with CPCs cultured under normoxic conditions (H0) (P < 0.01) (Fig. 2a). These data suggest that HP of c-kit (+) CPCs could activate the PI3K/Akt pathway.

To investigate whether HP could inhibit apoptosis and improve cell survival via the PI3K/Akt pathway, we measured the viability of c-kit (+) CPCs by flow cytometry. The apoptosis rate of CPCs in the H6+24 h group (15.05 ± 1.54%) was obviously reduced compared with the H0+24 h group (28.82 ± 1.43%) (P < 0.05). LY294002 (a specificPI3k inhibitor) almost completely inhibited the pro-survival effect of HP on CPCs in the H6+24 h group (31.07 ± 1.44%), whereas insulin-like growth factor-1 (IGF-1, a PI3K-specific agonist) decreased the CPC apoptosis rate in the H0+24 h group (17.26 ± 2.13%) (Fig. 2b). an IGF-1 concentration of 100 ng/ml in complete media could activate p-Akt enough to play a role in the cytoprotective effects on CPCs. LY294002, at 50 μM, could almost block the IGF-1 effect but not completely (Supplementary Figure S1). To further confirm the levels of apoptosis, caspase 3 protein expression levels were analysed by Western blot, and the results indicated that cleaved caspase 3 protein expression was decreased significantly in the H6+24 h group compared with that in the H0+24 h group (P < 0.01) (Fig. 2c). IGF-1 also inhibited cleaved caspase 3 expression, whereas LY294002 up-regulated its expression in hypoxia-preconditioned CPCs. In addition, the MTT assay was used to assess cell viability, and an analogous trend was evident among the six groups (Fig. 2d). These data suggest that the PI3K/Akt pathway plays a pivotal role in the cytoprotective effects of HP.

### Hypoxic preconditioning represses p53 and up-regulates DNA methyltransferases via the PI3K/Akt pathway

**Figure 3:**
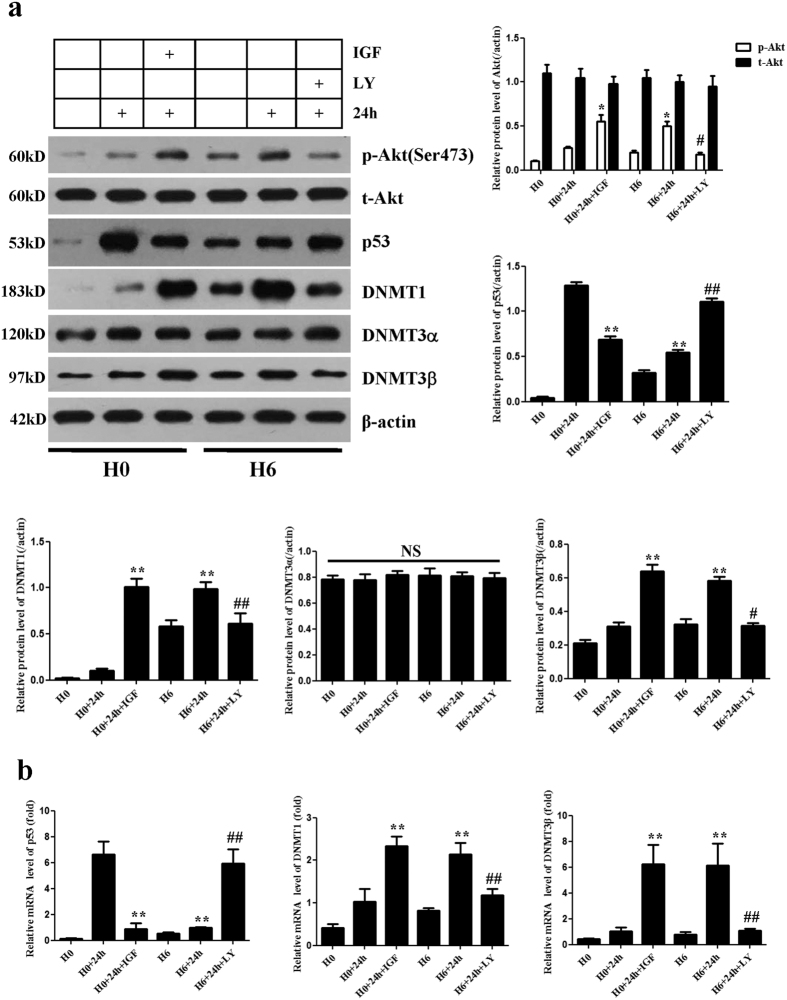
Hypoxic preconditioning represses p53 and up-regulates DNA methyltransferases via the PI3K/Akt pathway. (**a**) Representative Western blots of p53, DNMT1, DNMT3α and DNMT3β protein expression in CPCs. β-Actin was used as a loading control. p53 expression was markedly reduced by HP. DNMT1 and DNMT3β expression levels were significantly up-regulated by HP. DNMT3α expression was unchanged in all 6 groups. (**b**) q-PCR analysis of the mRNA expression of p53, DNMT1 and DNMT3β. Relative gene expression levels were calculated via normalization to GAPDH. HP reduced the mRNA level of p53 and up-regulated DNMT1 and DNMT3β. Data were obtained from three independent experiments under the same experimental conditions and are expressed as the mean ± SD. *P < 0.05 vs. H0 + 24 h. **P < 0.01 vs. H0+24 h. ^#^P < 0.05 vs. H6+24 h. ^##^P < 0.01 vs. H6+24 h. H0, normoxia. H0+24 h, normoxia + oxygen–serum deprivation for 24 hours. H0+IGF+24 h, normoxia with IGF-1 + oxygen–serum deprivation for 24 hours. H6, HP for 6 hours. H6+24 h, HP for 6 hours + oxygen–serum deprivation for 24 hours. H6+LY+24 h, HP for 6 hours with LY294002 + oxygen–serum deprivation for 24 hours. NS, not significant.

Given that p53 is the pivotal pro-apoptotic gene in cells, we next investigated whether HP of CPCs could influence p53 expression. Western blot and quantitative real-time polymerase chain reaction (PCR) confirmed that p53 mRNA and protein were highly expressed in the H0+24 h group. However, p53 mRNA and protein expression was obviously reduced in the H6+24 h group compared with the H0+24 h group (P < 0.01) (Fig. 3a,b). p53 expression was also largely reduced when IGF-1 was added to the medium of the H0+24 h group, whereas p53 expression was restored when c-kit (+) CPCs in the H6+24 h group were cultured with LY294002 (Fig. 3a,b).

DNMTs play an important role in modulating hypoxia-induced fibrosis within the heart^19^. To determine whether HP of CPCs modulates the expression of DNMTs, we next investigated DNMTs protein and mRNA levels. Western blot and quantitative real-time PCR (qPCR), respectively, revealed that DNMT1 and DNMT3β protein and mRNA expression levels were both significantly up-regulated in the H6+24 h group compared with the H0+24 h group (P < 0.01) (Fig. 3a,b), but that DNMT1 and DNMT3β protein and mRNA expression levels decreased sharply when LY294002 was added to the medium of the H6+24 h group. Culturing CPCs in the H0+24 h group with IGF-1 restored DNMT1 and DNMT3β protein and mRNA expression levels. Interestingly, DNMT3α protein expression was unaltered in all 6 groups (P > 0.05) (Fig. 3a). p-Akt expression showed a consistent trend similar to that of DNMT1 and DNMT3β (Fig. 3a). These data suggest that HP represses p53 and up-regulates DNMT1 and DNMT3β via the PI3K/Akt pathway.

### Both the targeted inhibition of DNMT1 and inhibition of DNMT catalytic activity induce p53 activation

**Figure 4:**
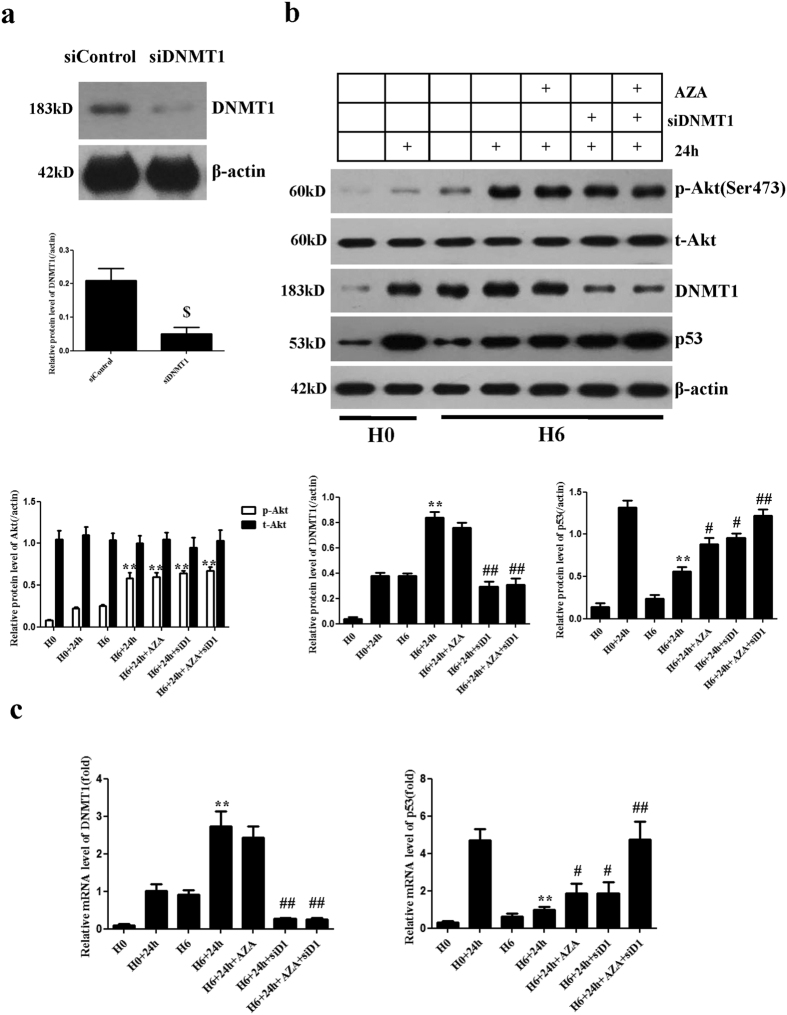
Targeted inhibition of DNMT1 induces p53 activation. (**a**) Representative Western blots of DNMT1 knocked down by siRNA-DNMT1. (**b**) Representative Western blots of DNMT1, p53, and Akt protein expression in CPCs. β-Actin was used as a loading control. CPCs transfected with siRNA-DNMT1 in the H6+24 h group exhibited increased p53 protein levels. p53 expression was significantly elevated when hypoxia-preconditioned CPCs were incubated with 5-azadc. Both 5-azadc and siRNA-DNMT1 completely restored p53 expression, resulting in no significant difference compared with the H0+24 h group. (**c**) q-PCR analysis of the mRNA expression of DNMT1 and p53. Relative gene expression levels were calculated via normalization to GAPDH. The trend was consistent with the observed protein expression levels. Data were obtained from three independent experiments under the same experimental conditions and are expressed as the mean ± SD. ^$^P < 0.01 vs. siControl. **P < 0.01 vs. H0+24 h. ^#^P < 0.05 vs. H6+24 h. ^##^P < 0.01 vs. H6+24 h. H0, normoxia. H0+24 h, normoxia + oxygen–serum deprivation for 24 hours. H6, HP for 6 hours. H6+24 h, HP for 6 hours + oxygen-serum deprivation for 24 hours. H6+AZA+24 h, HP for 6 hours with 5-azadc (a DNA methyltransferases inhibitor) + oxygen–serum deprivation for 24 hours. H6+siD1+24 h, HP for 6 hours with siRNA-DNMT1 + oxygen–serum deprivation for 24 hours. H6+AZA+siD1+24 h, HP for 6 hours with 5-azadc and siRNA-DNMT1 + oxygen–serum deprivation for 24 hours.

Previous studies have reported that the *de novo* methylating enzyme DNMT3β is the most likely candidate responsible for hypoxia-induced aberrant DNA hypermethylation^19^. Furthermore, several studies in recent years have indicated that a loss of DNMT1 increases the expression of p53, which does not require its catalytic activity^20^,^21^. To investigate whether a loss of DNMT1 reduced p53 expression in hypoxia-preconditioned CPCs, we assessed DNMT1 and p53 protein and mRNA expression levels by Western blot and qPCR, respectively. DNMT1 was successfully knocked down after transfection with siRNA-DNMT1 (Fig. 4a). siRNA-DNMT1-transfected CPCs in the H6+24 h group exhibited increased p53 protein and mRNA expression levels compared with cells in the H6+24 h group without transfection (P < 0.05) (Fig. 4b,c). When CPCs in the H6+24 h group were incubated with 5-aza-2′-deoxycytidine (5-azadc), a DNA methyltransferase inhibitor that can block the catalytic activity of DNMTs, DNMT1 expression was slightly reduced without obvious significance compared with the H6+24 h group (P > 0.05), whereas p53 expression was significantly elevated (P < 0.05) (Fig. 4b,c). When CPCs in the H6+24 h group were treated with both 5-azadc and siRNA-DNMT1, p53 protein and mRNA expression levels were completely restored and exhibited no significant difference compared with the H0+24 h group (P > 0.05) (Fig. 4b,c). These data suggest that both the loss of DNMT1 and inhibition of DNMT catalytic activity could activate p53.

### Catalytic activity of DNMTs does not induce p53 promoter hypermethylation

**Figure 5:**
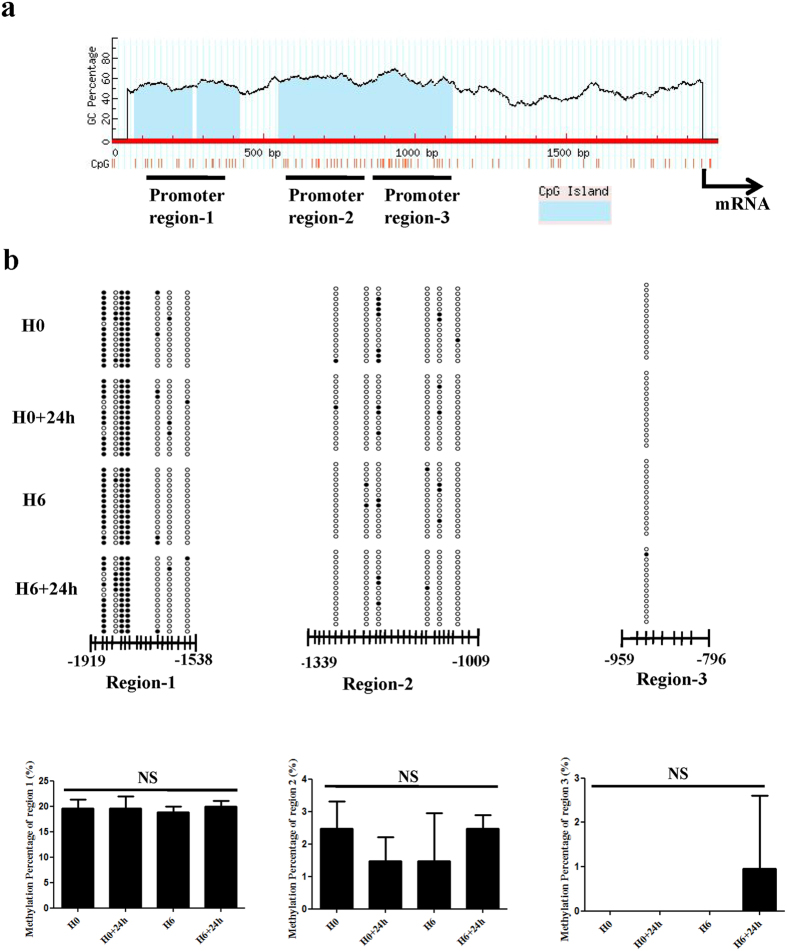
Catalytic activity of DNMTs does not induce p53 promoter hypermethylation. (**a**) Schematic diagram of the p53 promoter region, referring to http://www.urogene.org/methprimer/. The p53 promoter contains 3 candidate CpG islands. (**b**) Bisulphite sequencing analysis was performed to quantify CpG methylation. The representative methylation status of CpG sites in all 3 promoter regions is presented; 15 single clones were sequenced for each group. Black and white squares represent the methylated and unmethylated CpG sites, respectively. (**c**) Quantitative analysis of methylation percentage revealed no statistically significant difference among four groups. H0, normoxia. H0+24 h, normoxia + oxygen–serum deprivation for 24 hours. H6, HP for 6 hours. H6+24 h, HP for 6 hours + oxygen–serum deprivation for 24 hours. NS, not significant.

To determine the possible mechanisms of p53 suppression, we investigated whether DNMTs could repress p53 by catalysing the hypermethylation of its promoter. We investigated the p53 promoter region encompassing the 2000 bp upstream of the p53 transcription start site and identified 3 candidate CpG islands in this region (Fig. 5a). Bisulphite sequencing analysis was performed to quantify the methylation status of the CpG islands within 3 samples in each group and 5 clones in each sample. Unexpectedly, only a few sites exhibited varying levels of methylation in all 3 promoter regions, and the remaining CpG sites were all unmethylated (Fig. 5b). Quantitative analysis revealed that the methylation levels in all 3 promoter regions were minimally altered in the H6+24 h group compared with the H0+24 h group (P > 0.05) (Fig. 5b). These results suggest that DNMTs that are up-regulated by the PI3K/Akt pathway do not hypermethylate the p53 promoter in hypoxia-preconditioned CPCs.

### DNMT1 contributes to the transcriptional inhibition of p53

**Figure 6:**
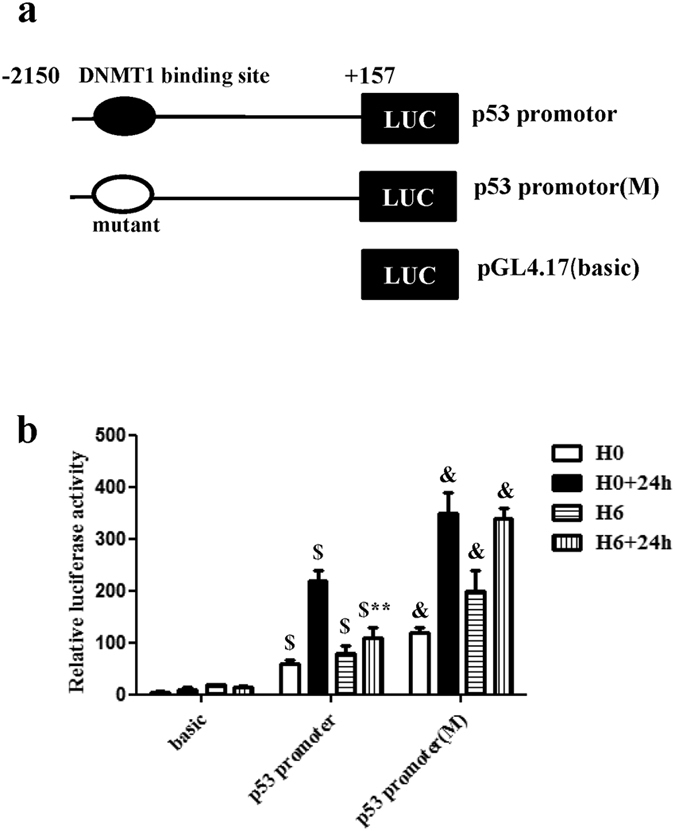
DNMT1 contributes to the transcriptional inhibition of p53. (**a**) Schematic diagram of the luciferase reporter constructs containing the indicated p53 gene promoter sequences. The putative DNMT1 binding site relative to the transcriptional initiation site of the p53 gene is indicated. (**b**) Quantitative analysis of relative luciferase activity. HP-treated samples in the p53 promoter group showed lower promoter activity than untreated samples. Promoter activities in the DNMT1-binding site mutant group increased significantly compared with those in the p53 promoter group. Data were obtained from three independent experiments under the same experimental conditions and are expressed as the mean ± SD. ^$^P < 0.01 vs. basic. ^&^P < 0.01 vs. p53 promoter. **P < 0.01 vs. H0 + 24 h. H0, normoxia. H0+24 h, normoxia + oxygen–serum deprivation for 24 hours. H6, HP for 6 hours. H6+24 h, HP for 6 hours + oxygen–serum deprivation for 24 hours.

To determine whether DNMT1 may contribute to transcriptional inhibition of the p53 gene through binding to the p53 promoter, a reporter gene assay was performed using the pGL4.17-basic vector containing the p53 promoter. The putative DNMT1 binding site, approximately 2k bp upstream of the p53 transcription start site^20^, was mutated to construct a mutated promoter (Fig. 6a). The results showed that relative luciferase activity was increased significantly in the p53 promoter group compared with the negative control (basic group) (P < 0.01) (Fig. 6b). Interestingly, we noted that HP-treated samples in the p53 promoter group showed lower promoter activity than untreated samples (P < 0.01) and that the degree of HP time dependency of promoter activity was consistent with the observed DNMT1 protein expression ratios (Figs 3a and 6b). We also observed a significant change in promoter activities in the DNMT1-binding site mutant group compared with the p53 promoter group (P < 0.01) (Fig. 6b). Thus, HP-induced over-expression of DNMT1 decreased p53 promoter activity. These data suggest that the above mentioned decreases in promoter activity may be due to direct binding between DNMT1 and the p53 promoter, which plays an important role in transcriptional inhibition of the p53 gene through the DNMT1 binding site.

### DNMT1 may directly repress p53 by binding to its promoter locus

**Figure 7:**
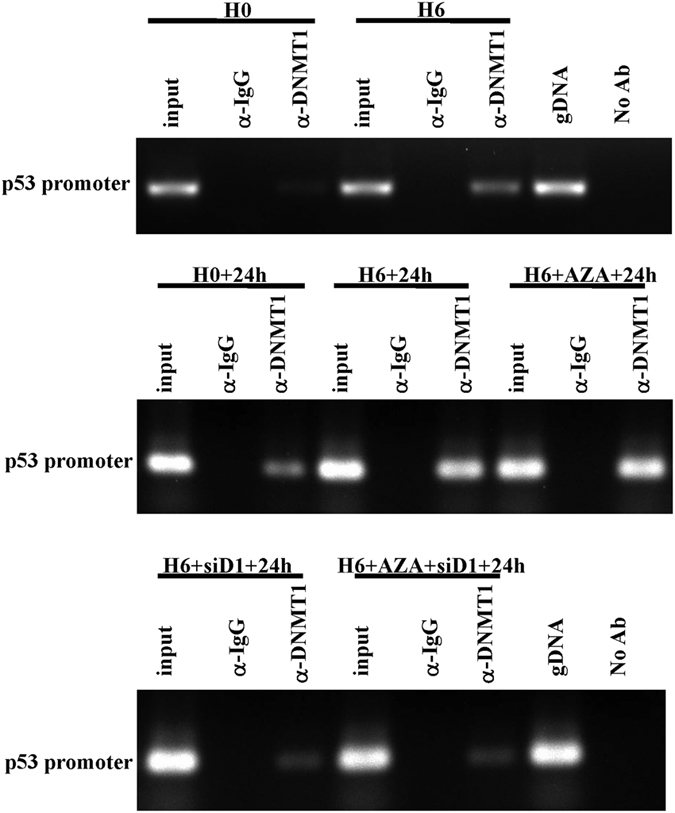
DNMT1 represses p53 by binding to its promoter locus. ChIP assay was used to further confirm DNMT1 binding activity in all seven groups. Chromatin was prepared from treated CPCs and immunoprecipitated using DNMT1 antibody. DNA sequence fragments from the p53 promoter onto which DNMT1 was recruited in all groups were amplified by PCR using specific primers. Data were obtained from three independent experiments under the same experimental conditions. Input, input chromatin prior to immunoprecipitation. α-IgG, immunoprecipitation with normal rabbit IgG. α-DNMT1, immunoprecipitation with DNMT1 antibody. gDNA, amplification of genomic DNA containing the p53 promoter sequence. No Ab, immunoprecipitation without antibody. H0, normoxia. H0+24 h, normoxia + oxygen–serum deprivation for 24 hours. H6, HP for 6 hours. H6+24 h, HP for 6 hours + oxygen–serum deprivation for 24 hours. H6+AZA+24 h, HP for 6 hours with 5-azadc (a DNA methyltransferases inhibitor) + oxygen–serum deprivation for 24 hours. H6+siD1+24 h, HP for 6 hours with siRNA-DNMT1 + oxygen–serum deprivation for 24 hours. H6+AZA+siD1+24 h, HP for 6 hours with 5-azadc and siRNA-DNMT1 + oxygen–serum deprivation for 24 hours.

A previous study by Georgia *et al*. demonstrated that DNMT1 binds to the p53 promoter approximately 2k bp upstream of the p53 transcription start site^20^. We hypothesized that DNMT1 might also play a role in directly repressing p53 transcription in hypoxia-preconditioned CPCs. Chromatin immunoprecipitation (ChIP) analysis was performed to further investigate whether DNMT1 could bind to the promoter region of p53 in hypoxia-preconditioned CPCs. As shown in Fig. 7, DNA sequence fragments from the p53 promoter onto which DNMT1 was recruited in all groups were amplified by PCR using specific primers. These results suggested that DNMT1 might directly repress the transcription of p53 by binding to its promoter locus.

## Discussion

The main findings of our investigation are as follows: (1) HP enhances the anti-apoptosis capacity of c-kit (+) CPCs and improves their survival via the PI3K/Akt pathway; (2) HP of c-kit (+) CPCs inhibits p53 by up-regulating DNMT1 and DNMT3β, which does not result in p53 promoter hypermethylation; and (3) DNMT1 might directly repressp53, at least in part, by binding to the p53 promoter locus.

It is widely accepted that the heart should no longer be regarded as a terminally differentiated organ. In the last decade, CPCs have been successfully acquired from the hearts of humans and other animals^3^,^22^. Among these CPCs, several subpopulations have been effectively separated by magnetic-activated cell sorting, such as c-kit (+) CPCs, Scal-1 (+) CPCs, side population cells, and cardiosphere-derived cardiac cells^23^. c-kit (+) CPCs are positive for c-kit and negative for haematopoietic lineage markers (most notably, CD34 and CD45). Beltrami *et al*. identified these cells as a population of multipotent, self-renewing clonogenic cells^3^. Furthermore, accumulating evidence has demonstrated that c-kit (+) CPCs exhibit enhanced proliferation and differentiation abilities to repair injured myocardium^24^. As shown in the present study, we successfully obtained highly purified c-kit (+) CPCs from adult mouse hearts through enzymatic digestion as well as magnetic-activated cell sorting.

Although c-kit (+) CPCs are the most likely candidates for cell therapy for congestive heart failure after AMI, poor survival of CPCs after engraftment remains the major obstacle for their clinical application. In recent years, many reports have indicated that the functional benefits of CPCs observed after transplantation might be related to the secretion of soluble factors that act in a paracrine fashion^25^. However, an adequate amount of transplanted CPCs is required not only for the generation of cardiomyocytes or vascular cells directly from CPCs but also for their protective paracrine effects; therefore, CPCs are subjected to various preconditioning stimuli to improve their survival. HP with sublethal hypoxic insult can enhance the survival and proliferation of stem cells *in vitro* and *in vivo* after transplantation^9^,^26^,^27^. Furthermore, our group has reported that HP of c-kit (+) CPCs for 6 hours is the optimal time course for protective effects^7^,^8^. Consistent with these investigations, our study provides additional evidence that HP enhances the anti-apoptotic capacity of c-kit (+) CPCs and improves their survival.

It has been well documented that constitutive activation of PI3K/Akt signalling is sufficient to block cell death induced by a variety of apoptotic stimuli. Previous investigations have demonstrated that the pro-survival function of Akt is activated by hypoxia, as a mediator of preconditioning signalling in various cell types^10^,^11^. Within our group, Yan *et al*. found that HP could improve the survival and retention of CPCs in ischaemic heart tissue by activating the SDF-1a/CXCR4 axis and the downstream anti-apoptosis pathway (the PI3K/Akt signalling pathway)^7^. In another study by our group, Hu *et al*. observed that Pim-1, which lies downstream of nuclear Akt accumulation, played a pivotal role in the protective effect of HP^8^. Up-regulation of Pim-1 by Akt caused a reduction of pro-apoptotic elements (cytochrome c and cleaved caspase-3) and preservation/modulation of important components of the mitochondria (Bcl-2,Bcl-XL and p-Bad), in addition to attenuating mitochondrial damage^8^. These studies by our group focused on molecules upstream and downstream of pro-survival signalling and the PI3K/Akt pathway, which may play a central role in cell survival in response to hypoxia. In the present study, we further found that the protective effects were almost completely inhibited by LY294002 and restored by IGF-1. Thus, IGF-1-mediated activation of the PI3K/Akt pathway plays a pivotal role in HP.

The primary target of HP is activation of survival mechanisms within the cell and stimulation of serial adaptive cellular responses to support the survival of CPCs in infarcted hearts after engraftment. A beneficial role of IGF-1 in cell regeneration has been extensively reported in other organs, whereas evidence of such effects in the heart has been limited. Administration of IGF-1 to c-kit (+) cells before transplantation into an infarcted myocardium enhances the recovery of cardiac function and myocardial remodelling^28^,^29^. Furthermore, local administration of IGF-1 or IGF-1 analogues to the heart could be useful for increasing the number of endogenous c-kit (+) cells after acute heart failure caused by AMI. Alternatively, exposure of c-kit (+) cells to exogenous IGF-1 before engraftment may represent a promising approach in patients with chronic heart failure. Despite the beneficial roles of IGF-1 in the cardiovascular system, the signalling pathways activated by IGF-1 in cardiac progenitors are poorly understood.

Given its ability to initiate cell cycle arrest and apoptosis during differentiation, p53 is a renowned pro-apoptotic tumour suppressor gene in cancer research. p53 is maintained at a low level in normal cells but becomes rapidly stabilized and activated in response to DNA damage, hypoxia, hyperproliferation, and other types of cellular stresses^30^. Lethal hypoxic insult of cells up-regulates p53 expression significantly and promotes apoptosis, whereas HP of cells prior to ischaemia insult represses p53 expression through activating hypoxia-inducible factor-1α^16^. Our investigation also found that HP down-regulates p53 by activating the PI3K/Akt pathway.

DNA methyltransferases (DNMTs) are responsible for DNA hypermethylation and cause gene silencing in mammalian cells. DNMT1 maintains the DNA methylation pattern and phenotype of dividing cells, whereas DNMT3α and DNMT3β are *de novo* methylating enzymes that are responsible for establishing the initial methylation patterns of the genome during development^18^. DNMTs play crucial roles in cell growth, differentiation and apoptosis in mammals. Recent reports have shown that deletion of DNMT1 results in rapid cell death in human embryonic stem cells^31^ and that abnormal methylation of CpG islands is induced by DNMTs in cardiovascular disorders and leads to changes in gene expression^32^. Watson *et al*. reported that hypoxia increases the expression of the DNA methyltransferases DNMT1 and DNMT3β, which play important roles in modulating hypoxia-induced fibrosis within the heart^19^. The modulation of DNMT enzymes and the resultant changes in DNA methylation mediated by hypoxia-inducible factor-1α may be important normal physiological mediators in the response to chronic hypoxia. Therefore, targeting up-regulated DNMT expression in ischaemic heart disease may prove to be a valuable therapeutic approach. Consistent with the above results, the findings of the present study indicate that the expression of both DNMT1 and DNMT3β in CPCs was significantly up-regulated by HP. These results imply that HP may repress p53 by up-regulating DNMT1 and DNMT3β.

As observed in cancer research, both hypo- and hyper-methylation gene-specific changes may occur within the same hypoxic environment. The DNA methylation status of p53 is unstable and is frequently impacted by many complicated factors, including interactions and crosslinks between p53 and its upstream or downstream genes. Cytosine hypermethylation silences normal gene expression in cells through epigenetic modifications that decrease p53 expression below functional levels. Hodge *et al*. reported that hypermethylation of the p53 promoter region, which was epigenetically modified by DNMT1, resulted in reduced levels of p53 expression in an IL-6-responsive multiple myeloma cell line^33^. Qing *et al*. reported that berberine could repress DNMT1 and DNMT3β expression, triggering hypomethylation of p53 by altering DNA methylation levels^34^. Additionally, a specially methylated residue (nucleotide −450) has been associated with transcriptional inactivation of the p53 gene^35^. DNA methylation of the p53 promoter may play a critical role in gene silencing in hypoxia-preconditioned cardiac progenitor cells. To confirm whether DNMTs can repress p53 by catalysing the hypermethylation of its promoter, we investigated the p53 promoter region encompassing the 2000 bp upstream of the p53 transcription start site and identified 3 CpG islands in this region. As indicated by our data, HP did not cause DNA hypermethylation of the entire p53 promoter region. Thus, the results of the present study indicate that DNMTs may play additional roles in p53 modulation, rather than catalysing p53 promoter hypermethylation.

Recently, several investigations have reported that DNMT1 directly represses p53 independent of its catalytic activity^20^,^21^. Similar results were obtained in our study, indicating that targeted inhibition of DNMT1 obviously up-regulated p53 instead of altering the p53 promoter methylation status. vAccordingly, these observations suggest that DNMT1 could have another role in repressing p53 without the need for its catalytic function. Furthermore, hypoxia-preconditioned CPCs incubated with 5-azadc expressed increased p53 levels compared with the control, but 5-azadc did not completely restore p53 levels. As a DNA methyltransferase inhibitor, 5-azadc not only inhibits the catalytic activity of DNMTs but also slightly degrades them. Thus, 5-azadc may activate p53 by inhibiting the catalytic activity of DNMTs, which did not catalyse DNA hypermethylation in the p53 promoter but might cause hypermethylation of other upstream or downstream genes of p53. DNMT1 may possess an additional function that is involved in the transcriptional repression mechanism in addition to DNA methyltransferase activity to provide multiple layers of gene silencing. When CPCs were simultaneously incubated with 5-azadc and siRNA-DNMT1, both the transcriptional repression mechanism and DNA methyltransferase activity of DNMT1 were inhibited. Hence, the protective effects were almost completely abolished.

Previous studies have found that DNMT1 represses p53 to maintain progenitor cell survival during pancreatic organogenesis and modulates gene expression without requiring its catalytic activity^20^. In addition, deletion of the catalytic domain of DNMT1 did not abolish the repressive activity of the protein against reporter genes^21^. We also found that DNMT1 might directly inhibit the transcription of p53 in hypoxia-preconditioned CPCs. We performed a reporter gene assay to verify that the up-regulation of DNMT1 by HP plays an important role in transcriptional inhibition of the p53 gene through the DNMT1 binding site. ChIP analysis further confirmed that DNMT1 could bind to the promoter locus of p53 in hypoxia-preconditioned CPCs, which implies that DNMT1 might directly repress the transcription of p53 by binding to its promoter locus. Furthermore, DNMT1 might act as a transcriptional repressor through a scaffolding function of the protein that recruits other transcriptional repressive complexes to target genes^21^. DNMT1 cooperates with a number of repressive proteins and histone-modifying enzymes in gene silencing. Georgia *et al*. reported that both DNMT1 and CTCF bind to the p53 regulatory region, indicating that cooperation between DNMT1 and CTCF is associated with p53 repression^20^. Clements *et al*. reported that DNMT1 modulates gene expression partially through its interactions with histone-modifying enzymes, such as LSD1^21^. Therefore, DNMT1 may exhibit multi-faceted functions that cooperate with repressive proteins or histone-modifying enzymes to provide multiple layers of gene silencing. The sites at which DNMT1 functions and the types of protein complexes in which it participates as a co-repressor will be the subjects of future studies.

Collectively, our study demonstrates that HP enhances the anti-apoptotic capacity of c-kit (+) CPCs and improves their survival via the PI3K/Akt pathway. p53 is down-regulated by DNMTs, which do not hypermethylate the promoter of p53 in hypoxia-preconditioned c-kit (+) CPCs. In addition, DNMT1 might directly repress p53 expression, at least in part, by binding to the p53 promoter locus. However, the mechanism underlying p53 modulation is extremely complicated and is cross-linked with many cell signalling pathways. For example, despite advances in other fields, the interaction between p53 and hypoxia-inducible factor-1α in hypoxia-preconditioned CPCs remains obscure and contradictory. This problem will be further studied in our next investigation.

## Methods

### Ethics Statement

All procedures in the present study were conducted in accordance with the National Institutes of Health Guide for the Care and Use of Laboratory Animals and approved by the Care of Experimental Animals Committee of Southeast University (approval ID: SYXK-2011.3923).

### Isolation and culture of CPCs

As previously described by our group^7^,^36^, CPCs were acquired from the hearts of two-month-old wild-type male C57BL/6 mice (Yangzhou Laboratory Animal Center). After the CPCs were cultured for approximately 10 days, a layer of fibroblast-like cells migrated from the adherent myocardial tissue. Numerous small, round, phase-bright cells emerged from these fibroblast-like cells. These cells were collected using the Accutase digestion enzyme, which does not influence surface markers. The acquired cells were further separated by magnetic-activated c-kit cell sorting magnetic beads (MiltenyiBiotec Inc., GER) according to the manufacturer’s instructions. These separated cells were seeded at 2 × 10^4^ cells/ml on poly-D-lysine-coated (Sigma, USA) dishes in cardiosphere growing medium (CGM: 65% DMEM-F12 [HyClone, USA] mixture containing 10% foetal calf serum [Gibco, USA], 2 mmol/l L-glutamine [HyClone, USA], 0.1 mmol/l 2-mercaptoethanol [Sigma, USA], 2% B27 [Gibco, USA], 5 ng/ml basic fibroblast growth factor (bFGF) [R&D, USA], 10 ng/ml epidermal growth factor (EGF) [Peprotech, USA], 40 nmol/l cardiotrophin-1 [Peprotech, USA], 1 unit/ml thrombin [Sigma, USA], 100 U/ml penicillin G [HyClone, USA], and 100 mg/ml streptomycin [HyClone, USA]).

### Characterization of CPCs

Phase-contrast microscopy was used to evaluate the morphology of CPCs, and flow cytometric analysis was performed to examine the expression of stem cell surface markers. CPCs were trypsinised, re-suspended in phosphate-buffered saline (PBS) and blocked with 3% FBS for 15 minutes for flow cytometric analysis. Next, CPCs were labelled with FITC-conjugated rat anti-mouse c-kit (BD Biosciences, USA), FITC-conjugated rat anti-mouse Scal-1 (BD Biosciences, USA), FITC-conjugated rat anti-mouse CD34 (MiltenyiBiotec Inc., GER) and FITC-conjugated rat anti-mouse CD45 (MiltenyiBiotec Inc., GER) at 4 °C in a dark room for 30 minutes and then washed twice with cold PBS. Mouse IgG1 antibody (BD Biosciences, USA) was used as an isotype control. Data were collected from 1 × 10^5^ cells using a FACS Calibur flow cytometer (BD Biosciences, USA) and analysed using WinMDI software.

### Hypoxic preconditioning of CPCs

Cells were incubated under hypoxic conditions in a Modular Incubator Chamber (Billumps-Rothenberg, Del Mar, CA) according to the manufacturer’s instructions. Briefly, the chamber was filled with a mixture of 0.1% O_2_, 5% CO_2_ and 94.9% N_2_ after CPCs were placed in it. The chamber was then closed tightly, and the cells were incubated at 37 °C for 6 hours. Next, all the cells were cultured continuously in RPMI-1640 medium without serum and exposed to 0.1% O_2_ with 5% CO_2_ and 94.9% N_2_ at 37 °C for 24 hours in the chamber (oxygen–serum deprivation, ischaemic cells). A special PI3K inhibitor, LY294002 (50 μM), and the PI3K-specific agonist, insulin-like growth factor 1 (100 ng/ml), were respectively added to the CPC medium prior to ischaemia.

### Assessment of cell viability

Cell viability was assessed using a MTT Cell Proliferation and Cytotoxicity Assay Kit (BeyotimeBiotech, China) in which the yellow MTT is reduced to purple formazan by mitochondrial dehydrogenase in live cells. Briefly, 10 μl of MTT solution (5 mg/ml) was added to each well followed by an additional 4 hours of incubation at 37 °C. The assay was stopped by the addition of 100 μl of dissolved formazan solution. Optical density (OD) was measured at 570 nm using an ELX-800 microplate assay reader (Bio-Tek, USA).

### Apoptosis assay

The apoptosis assay was performed using an Annexin V-FITC Kit (MiltenyiBiotec Inc., GER) according to the manufacturer’s instructions. Briefly, after the CPCs were cultured under hypoxic conditions for 24 hours, they were collected, washed, and resuspended in 100 μl of annexin V binding buffer. The cells were incubated with 10 μl of annexin V-FITC per 10^6^ cells for 15 minutes at room temperature in the dark. Next, the cell suspension was supplemented with 500 μl of binding buffer and 5 μl of propidium iodide per 10^6^ cells prior to flow cytometric analysis.

### Western blot analysis

The treated cells in the experiment were lysed with lysis buffer, and the cell extract protein concentration was quantified via a bicinchoninic acid assay. Western blot analysis was performed as follows. Equal amounts of the lysate proteins (40 μg) were denatured in 2× SDS-PAGE sample buffer and electrophoresed for 3 hours at 20 mA on 10% polyacrylamide gels. The separated proteins were then transferred onto polyvinylidene difluoride (PVDF) membranes, which were blocked by TBST solution (10 mMTris-HCl, 150 mMNaCl, and 0.05% Tween-20) containing 5% non-fat dry milk for 4 hours at room temperature. Next, the proteins were incubated with primary antibodies (Abcam, 1:1000 dilution) and then placed on a rocker at 4 °C overnight. Subsequently, after the membranes were washed three times with TBST for 15 minutes, the proteins were incubated with a horseradish peroxidase-conjugated secondary antibody (Santa Cruz, 1:5000 dilution) for 2 hours at room temperature and then washed thrice with TBST for 15 minutes. β-Actin was used as loading control (Santa Cruz, 1:1000 dilution). Immunoreactive bands were visualized using an enhanced chemiluminescence reagent, imaged using a five-minute exposure film and quantified by scanning densitometry.

### Quantitative real-time PCR

TRIzol reagent (Invitrogen, CA) was used to extract total RNA from the cells according to the manufacturer’s instructions. First-strand cDNA was acquired by reverse transcription using a cDNA synthesis kit (Fermentas, CA) according to the manufacturer’s instructions. The cDNA was stored at −20 °C until use. qPCR was performed using IQ SYBR Green Supermix (Bio-Rad, USA) and the Bio-Rad MJ Mini Opticon Real-Time PCR System along with the matching analysis software Bio-Rad CFX Manager. The resulting amplification and melt curves were analysed to ensure the identity of the specific PCR product. Relative gene expression levels were calculated by normalization to GAPDH. The sequence of each designed primer is as follows:

Mouse GAPDH primer

Sense: 5′-ACAACTTTGGCATTGTGGAA-3′,

Antisense: 5′-GATGCAGGGATGATGTTCTG-3′;

Mouse DNMT1 primer

Sense: 5′-CTGCTGTGGAGAAACTGGAA-3′,

Antisense: 5′-TGATTTCCGCCTCAATGATA-3′;

Mouse DNMT3β primer

Sense: 5′-CTCAAACCCAACAAGAAGCA-3′,

Antisense: 5′-AGCAGCAGAGTCATTGGTTG-3′;

Trp53 primer

Sense: 5′-CTCAGACTGACTGCCTCTGC-3′,

Antisense: 5′-GGCTGAGCCCTAGCTACAAG-3′.

### Bisulphite sequencing analysis

Genomic DNA was extracted from the cultured cells using a Genomic DNA Mini Preparation Kit with a Spin Column (Beyotime Biotech, China) according to the manufacturer’s instructions. DNA concentration and purity were determined based on the absorbance at 260 and 280 nm. A total of 1 μg of genomic DNA from each sample was bisulphite-treated using anEZ-96 DNA methylation kit (Zymo Research) according to the manufacturer’s instructions. Bisulphite sequencing was performed as previously described^37^. PCR primers were designed using Methprimer (http://www.urogene.org/methprimer/) as follows:

Trp53-Region1 Sense: 5′-TATTTATTATTTGTAATTTTTTAAGAAGTT-3′;

Trp53-Region1 Antisense: 5′-CTTCCAATAAATTAAATCCTAAAATC-3′.

Trp53-Region2 Sense: 5′-AGGGAGGTTATTYGGAGTTAAGAG-3′;

Trp53-Region2 Antisense: 5′-AAATATAAACAAAATCRTTCCTTCC-3′.

Trp53-Region3 Sense: 5′-TGTAGTTTGAATTTTGGGTTTTG-3′;

Trp53-Region3 Antisense: 5′-TCACAACCTTTAATTACACAACAAC-3′.

### siRNA-targeted DNMT1 knock-down

CPCs were transfected with either siDNMT1 or control siRNA using Lipofectamine RNAiMAX transfection reagent (Invitrogen, CA). The siRNA gene sequences used to specifically target DNMT1 were: sense: 5′-CCGAAGAUCAACUCACCAATT-3′ and antisense: 5′-UUGGUGAGUUGAUCUUCGGTT-3′. siControl and siDNMT1 were purchased from GenePharma (China). Transfected cells were incubated for 6 hours, replenished with fresh cell culture medium and assessed for changes in protein expression 48 hours later via Western blotting.

### Construction of p53 promoter luciferase reporter plasmids

A 2308 bp fragment (nucleotides from −2150 to +157) of the mouse p53 promoter was prepared through PCR amplification of mouse genomic DNA from CPCs using a sense primer containing an *Xho*I restriction site and an antisense primer containing a *Bgl*II restriction site. Primers were synthesized based on the reported genomic sequence of mouse p53 (sense: 5′-CCGCT CGAGC TGGAA CTCAC ACATC TGCCT GC-3′, and antisense: 5′-GGAAG ATCTC CAGTC TTCGG AGAAG CGTGA CA-3′). After the fragment was digested with restriction enzymes, the p53 promoter fragment was directionally cloned into the pGL4.17-basic firefly luciferase expression vector (Promega) to generate a p53 reporter plasmid. To prepare a mutated promoter, the putative DNMT1 binding site sequence GGGC was mutated to ATAT. All of the constructs were verified through sequencing.

### Dual-luciferase assay for promoter activity

The dual-luciferase assay was performed as described previously with some modifications^38^. Briefly, cells were plated in 24-well plates, cultured overnight, and transfected using Lipofectamine transfection reagent (Invitrogen) according to the manufacturer’s protocol. To normalize luciferase activity, the pRL-TK control vector encoding *Renilla* luciferase was co-transfected with the pGL4.17 plasmids. After 12 hours, HP of CPCs was performed for 6 hours. The cells were then cultured for 48 hours after transfection and lysed with passive lysis buffer (Promega). The lysates were analysed using a Dual-Luciferase Reporter Assay System Kit (Promega). Luminescence was measured on a luminometer (Turner Biosystems Instrument, USA). All experiments were performed at least three times.

### Chromatin immunoprecipitation

Cells were cross-linked as previously described^38^. Nuclear extracts from1 × 10^6^ cells were used per immunoprecipitation (IP). Cross-linked pellets were resuspended with CEBN. The solution was incubated on ice for 10 minutes with vortexing every minute, and the nuclear pellet was centrifuged at 6400 rpm in the cold for 5 minutes. The nuclear pellets were washed once with CEB and then resuspended with SDS lysis buffer (Millipore). Samples were sonicated to 200 bp to 1 kb fragments. Then, 60 to 80 mg of chromatin was used per IP, and appropriate amounts of antibodies (between 2 and 10 μg) were added to the sonicated DNA. Dynal magnetic beads (Invitrogen) were added for 3 hours, and wash conditions were adjusted based on the antibodies. IP-specific products were amplified by PCR. The PCR primer sequences were: sense: 5′-AACACGGTGGTGCGATACCAAG-3′ and antisense: 5′-CCAACACGGGCCCCTAAGTTC-3′.

### Statistical analysis

Statistical analysis was performed using SPSS (v 11.5, SPSS Inc.). The results are presented as the mean ±  standard deviation (SD) for normally and non-normally distributed continuous variables of at least three independent experiments unless otherwise stated, and frequencies and percentages summarize categorical variables. Statistical analysis of the differences between two groups was performed using Student’s two-tailed t-test, and differences between three or more groups were analysed using one-way analysis of variance (ANOVA) and Bonferroni multiple comparison. Statistical significance was defined as P < 0.05.

## Additional Information

**How to cite this article**: Xu, R. *et al*. Hypoxic Preconditioning Inhibits Hypoxia-induced Apoptosis of Cardiac Progenitor Cells via the PI3K/Akt-DNMT1-p53 Pathway. *Sci. Rep.* **6**, 30922; doi: 10.1038/srep30922 (2016).

## Supplementary Material

Supplementary Information
