# Supplementary material for: Hypoxic Preconditioning Inhibits Hypoxia-induced Apoptosis of Cardiac Progenitor Cells via the PI3K/Akt-DNMT1-p53 Pathway
Source: Sci Rep. 2016 Aug 4;6:30922. doi: 10.1038/srep30922 (PMC4973228; doi:10.1038/srep30922)
Supplement: Supplementary Information [file srep30922-s1.pdf]

Hypoxic Preconditioning Inhibits Hypoxia-induced Apoptosis of Cardiac Progenitor Cells via the PI3K/Akt-DNMT1-p53 Pathway

Authors:

Rongfeng Xu, Yuning Sun, Zhongpu Chen, Yuyu Yao & Genshan Ma*.

Affiliations:

Department of Cardiology, Zhongda Hospital, Medical School of Southeast University, Nanjing 210009, Jiangsu, China.

*Corresponding author:

Genshan Ma, MD, PhD, Professor; Department of Cardiology, Zhongda Hospital, Medical School of Southeast University, DingjiaQiao No.87, Hunan Road, Nanjing 210009, Jiangsu, China.

E-mail: magenshan@hotmail.com

SUPPLEMENTARY INFORMATION includes:

Supplementary Figures S1.

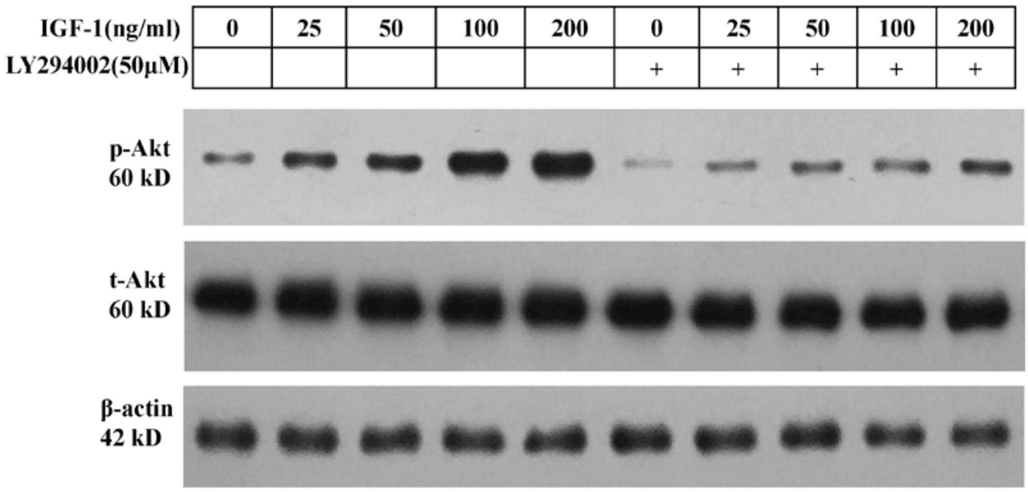

Supplementary Figure S1: **Representative Western blots of Akt activated by IGF-1 in CPCs.** β-actin was used as a loading control. an IGF-1 concentration of 100 ng/ml in complete media could activate p-Akt enough to play a role in the cytoprotective effects on CPCs. When we increased the IGF-1 concentration gradually, the p-Akt protein expression level did not increase accordingly. LY294002, at 50 μM, could almost block the IGF-1 effect but not completely.
